# Supplementary material for: A transcriptional network governing ceramide homeostasis establishes a cytokine-dependent developmental process
Source: Nat Commun. 2023 Nov 9;14:7262. doi: 10.1038/s41467-023-42978-w (PMC10636182; doi:10.1038/s41467-023-42978-w)
Supplement: Supplementary file 1 — Supplementary Information [file 41467_2023_42978_MOESM1_ESM.pdf]

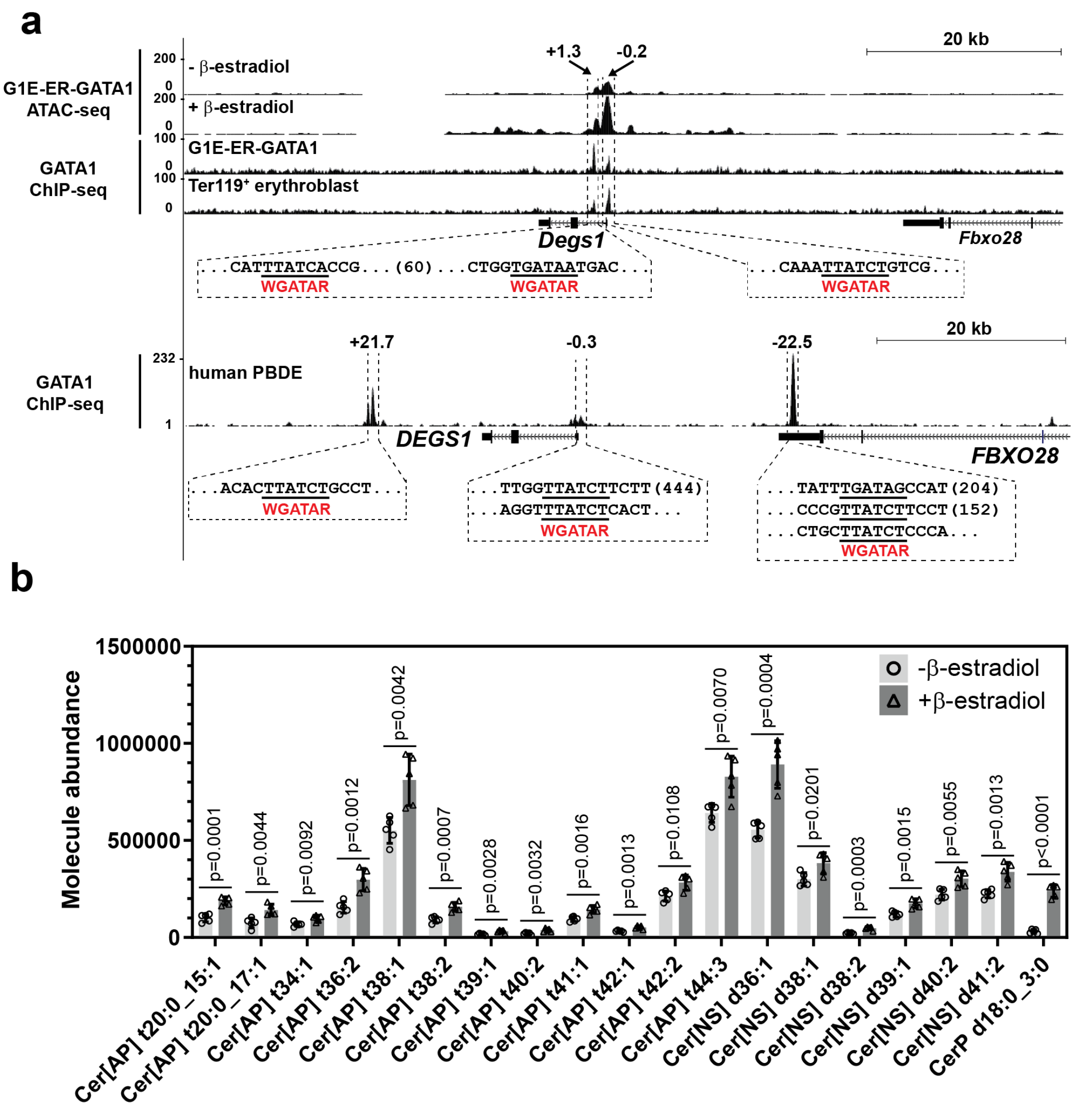

Supplementary Fig. 1. GATA1 controls ceramide homeostasis by regulating sphingolipid synthetic enzymes. Related to Fig. 1.

(a) ATAC-seq and GATA1 ChIP-seq profiles near murine *Degs1* and human *DEGS1* locus. WGATAR motifs in GATA1-occupied sequences are highlighted. PBDE: peripheral blood-derived erythroblasts.

(b) Discovery lipidomics revealed that multiple species of ceramide were upregulated during erythroid differentiation (n=5 biologically independent samples, mean ± SEM). p-values were calculated by two-tailed unpaired Student’s t-test. Source data are provided in Supplementary Data 2.

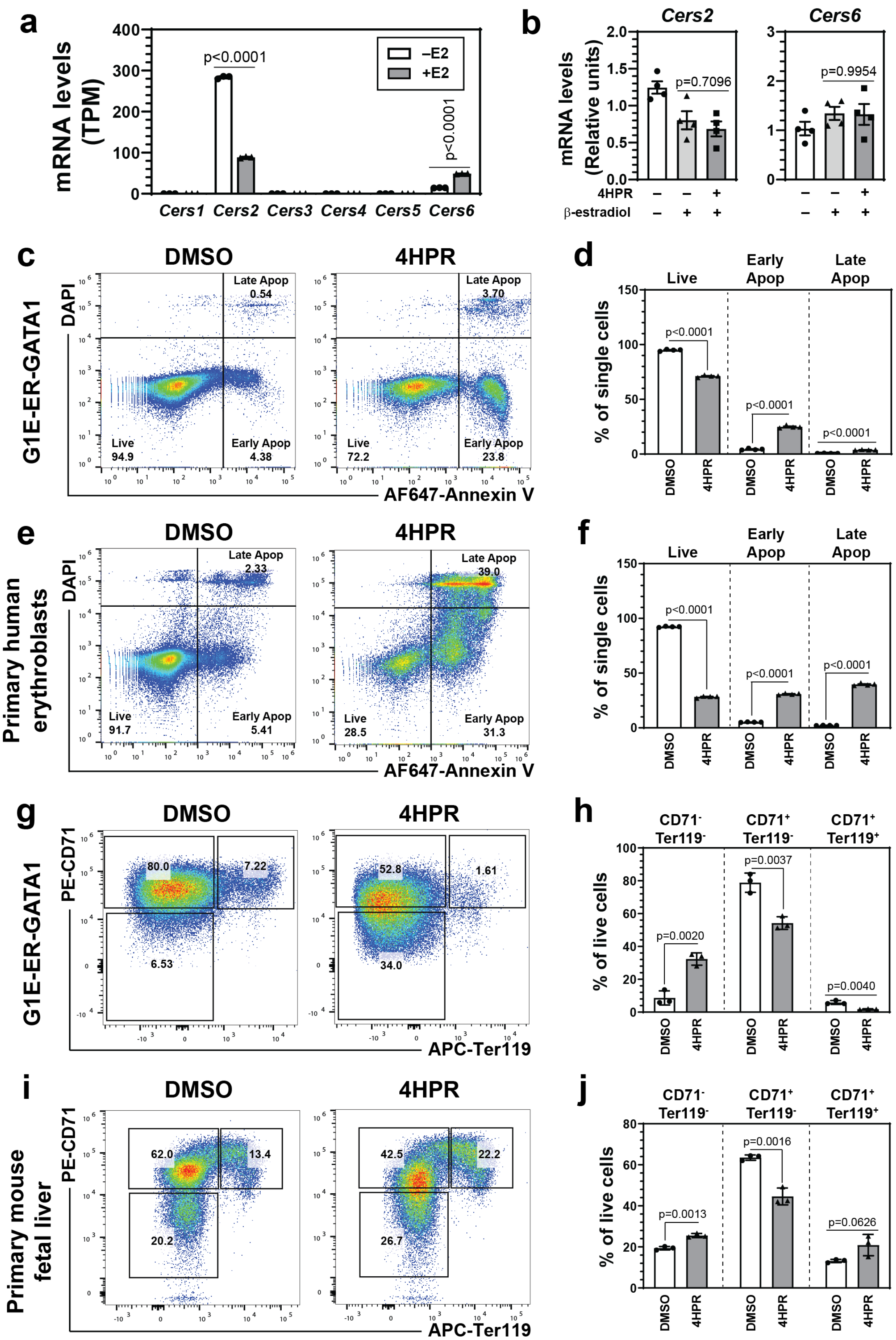

a
mRNA levels (TPM)
p<0.0001
p<0.0001
−E2
+E2
Cers1 Cers2 Cers3 Cers4 Cers5 Cers6
b
Cers2
Cers6
mRNA levels (Relative units)
p=0.7096
p=0.9954
4HPR
β-estradiol
c
DMSO
4HPR
G1E-ER-GATA1
DAPI
AF647-Annexin V
Late Apop 0.54
Live 94.9
Early Apop 4.38
Late Apop 3.70
Live 72.2
Early Apop 23.8
d
Live
Early Apop
Late Apop
% of single cells
p<0.0001
p<0.0001
p<0.0001
e
Primary human erythroblasts
Late Apop 2.33
Live 91.7
Early Apop 5.41
Late Apop 39.0
Live 28.5
Early Apop 31.3
f
g
PE-CD71
APC-Ter119
80.0
7.22
6.53
52.8
1.61
34.0
h
CD71- Ter119-
CD71+ Ter119-
CD71+ Ter119+
% of live cells
p=0.0020
p=0.0037
p=0.0040
i
Primary mouse fetal liver
62.0
13.4
20.2
42.5
22.2
26.7
j
p=0.0013
p=0.0016
p=0.0626

Supplementary Fig. 2. DES inhibition promotes apoptosis and inhibits differentiation of erythroid cells. Related to Fig. 2.

(a) mRNA expression of *Cers* genes was quantified by RNA-seq in G1E-ER-GATA1 cells treated with or without β-estradiol (E2) (n=3 biologically independent samples, mean ± SEM). p-values were calculated by two-tailed unpaired Student’s test.

(b) G1E-ER-GATA1 cells were differentiated with β-estradiol and treated with or without 4HPR for 24 h. mRNA levels of *Cers2* and *Cers6* were quantified by RT-qPCR (n=4 biologically independent samples, mean ± SEM). p-values were calculated using one-way ANOVA followed by Tukey’s multiple comparisons test.

(c) G1E-ER-GATA1 cells were treated with or without 4HPR for 24 h. Representative flow cytometry plots of Annexin V and DAPI were shown.

(d) Quantitation of the percentage of live, early apoptotic, and late apoptotic cells in (c) (n=4 biologically independent samples, mean ± SEM). p-values were calculated by two-tailed unpaired Student’s test.

(e) Primary human G-CSF-mobilized mononuclear cells were differentiated towards the erythroid lineage. D9 cells were treated with or without 4HPR for 48 h. Representative flow cytometry plots of Annexin V and DAPI were shown.

(f) Quantitation of the percentage of live, early apoptotic, and late apoptotic cells in (e) (n=4 biologically independent samples, mean ± SEM). p-values were calculated by two-tailed unpaired Student’s test.

(g) G1E-ER-GATA1 cells were treated with or without 4HPR and differentiated with β-estradiol for 48 h. Representative flow cytometry plots of CD71 and Ter119 were shown.

(h) Quantitation of the percentage of $CD71^-Ter119^-$, $CD71^+Ter119^-$, and $CD71^+Ter119^+$ populations out of total live cells from (g) (n=3 biologically independent samples, mean ± SEM). p-values were calculated by two-tailed unpaired Student’s test.

(i) Lin^- cells from E14.5 mouse fetal liver were treated with or without 4HPR for 48 h. Representative flow cytometry plots of CD71 and Ter119 were shown.

(j) Quantitation of the percentage of $CD71^-Ter119^-$, $CD71^+Ter119^-$, and $CD71^+Ter119^+$ populations out of total live cells from (i) (n=3 biologically independent samples, mean ± SEM). p-values were calculated by two-tailed unpaired Student’s test.

Source data are provided as a Source Data file.

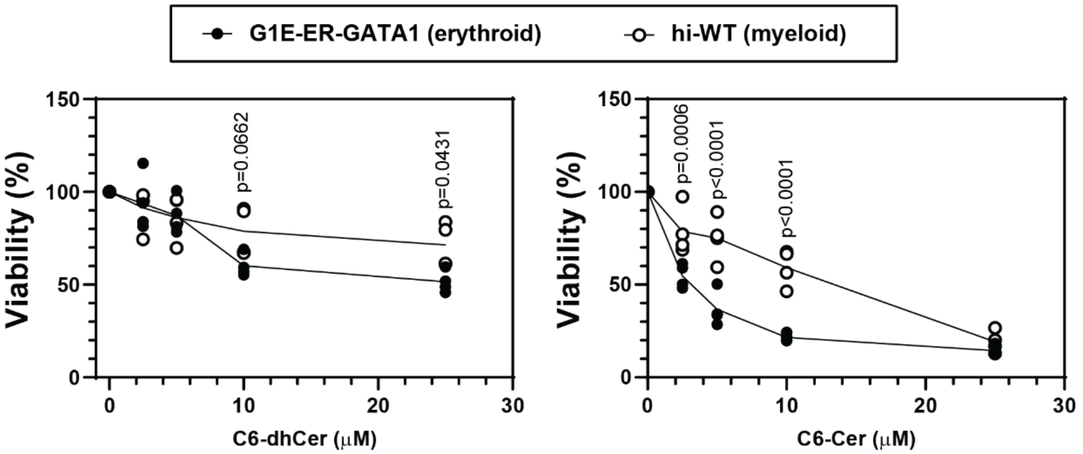

Supplementary Fig. 3. Disrupting ceramide homeostasis differentially impacts erythroid and myeloid cells. Related to Fig. 3.
G1E-ER-GATA1 (erythroid) and hi-WT (HOXB8-immortalized myeloid progenitor cells) were treated with increasing amount of C6-dhCer or C6-Cer for 24 h. Live cell numbers were quantified by Trypan blue dye exclusion assay. Viability was calculated by normalizing live cell numbers of (dh)Cer-treated samples to vehicle-treated ones for each cell type (n=4 biologically independent samples, mean ± SEM). p-values were calculated using two-way ANOVA followed by Sidak's multiple comparisons test.
Source data are provided as a Source Data file.

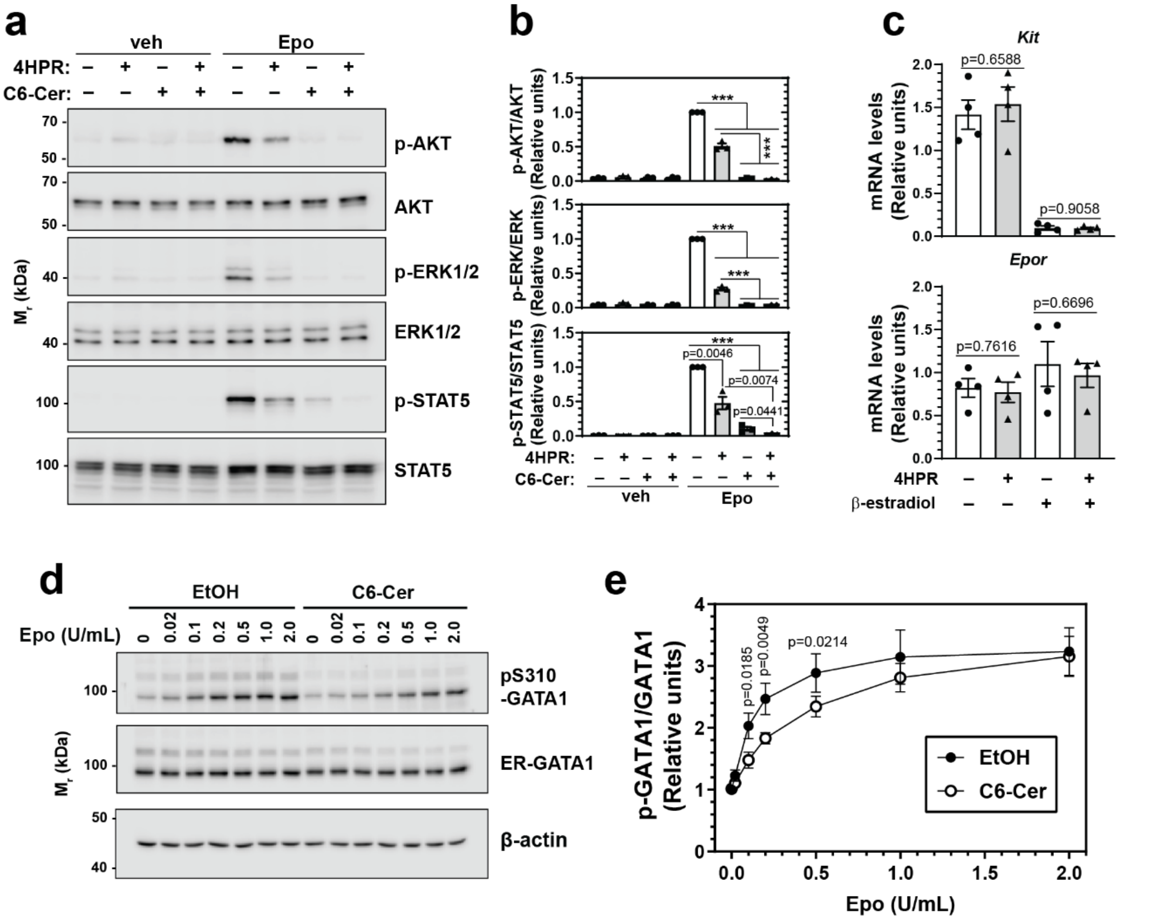

Supplementary Fig. 4. Ceramide homeostasis disruption inhibits cytokine-dependent signaling and transcription factor targeting. Related to Fig. 4.

(a) G1E-ER-GATA1 cells were treated with 4HPR and/or C6-Cer for 4 h and stimulated with Epo. Western blotting was performed to measure Epo signaling.

(b) Quantitation of blots in (a). Phosphorylation signals were normalized to their corresponding protein levels (n=3 independent experiments, mean ± SEM). p-values were calculated by two-tailed unpaired Student's test. ***p<0.0001.

(c) G1E-ER-GATA1 cells were treated with or without β-estradiol for 24 h followed by vehicle or 4HPR treatment for 4 h. mRNA levels of *Kit* and *Epor* were quantified by RT-qPCR (n=4 biologically independent samples, mean ± SEM). p-values were calculated by two-tailed unpaired Student's test.

(d) G1E-ER-GATA1 cells were Epo-starved overnight, treated with vehicle (EtOH) or C6-Cer for 2h, and stimulated with increasing amount of Epo for 2h. GATA1 phosphorylation at S310 was assessed by Western blotting.

(e) Quantitation of blots in (d). pS310-GATA1 signals were normalized to ER-GATA1 (n=6 independent experiments, mean ± SEM). p-values were calculated using two-way ANOVA followed by Sidak's multiple comparisons test.

Source data are provided as a Source Data file.

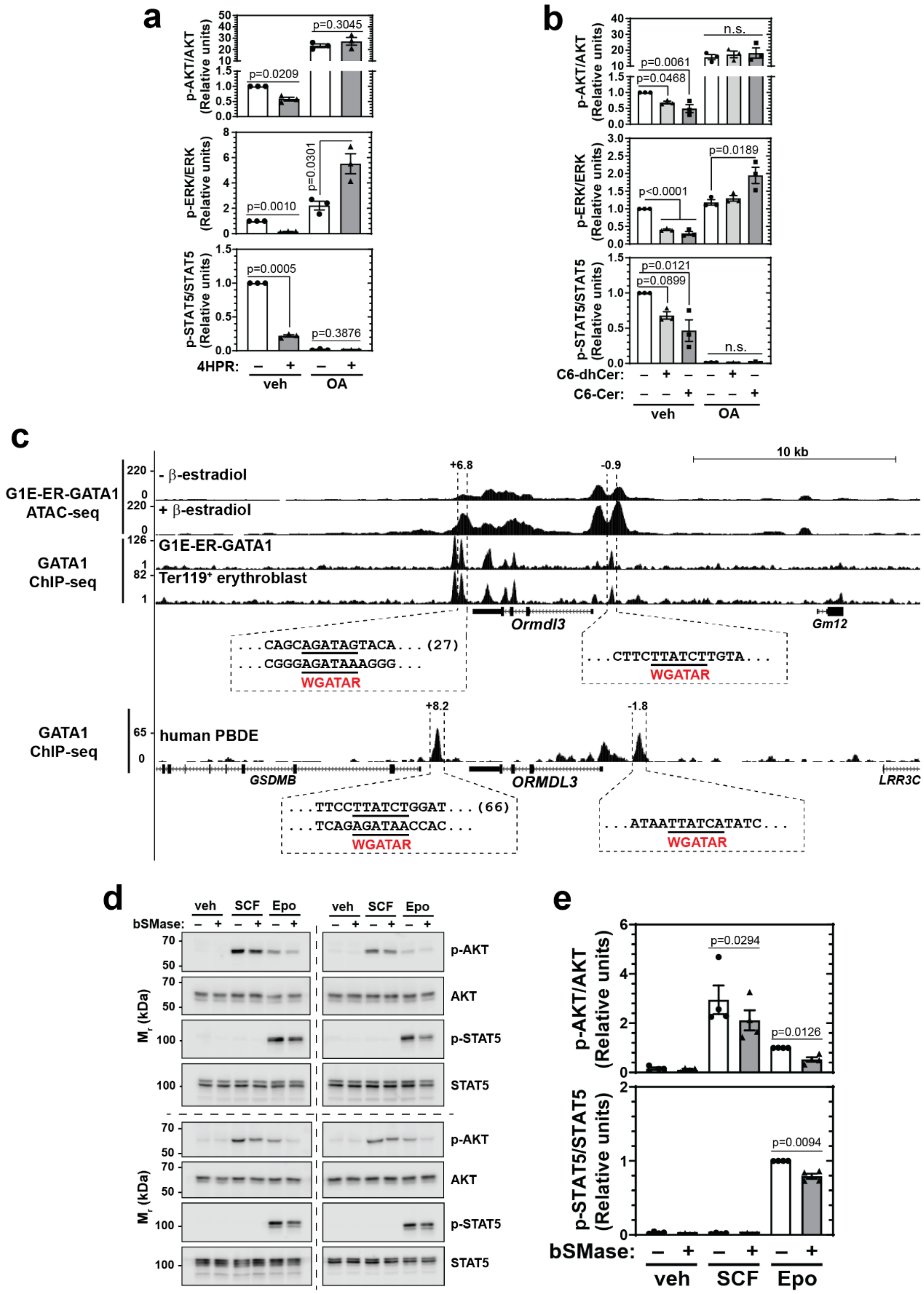
a
b
c
d
e
p-AKT/AKT (Relative units)
p-ERK/ERK (Relative units)
p-STAT5/STAT5 (Relative units)
4HPR:
veh
OA
C6-dhCer:
C6-Cer:
n.s.
G1E-ER-GATA1 ATAC-seq
- β-estradiol
+ β-estradiol
GATA1 ChIP-seq
G1E-ER-GATA1
Ter119+ erythroblast
Ormdl3
Gm12
10 kb
WGATAR
human PBDE
GSDMB
ORMDL3
LRR3C
bSMase:
SCF
Epo
p-AKT
AKT
p-STAT5
STAT5
Mr (kDa)

Supplementary Fig. 5. Manipulating endogenous ceramide homeostasis impairs cytokine signaling. Related to Fig. 5.

(a) Quantitation of blots in Fig. 5a. Phosphorylation signals were normalized to their respectively protein levels (n=3 biologically independent samples, mean ± SEM). p-values were calculated by two-tailed paired Student's test.

(b) Quantitation of blots in Fig. 5b. Phosphorylation signals were normalized to their respectively protein levels (n=3 biologically independent samples, mean ± SEM). p-values were calculated using one-way ANOVA followed by Dunnett's multiple comparisons test. n.s., not significant.

(c) ATAC-seq and GATA1 ChIP-seq profiles near murine *Ormdl3* and human *ORMDL3* locus. WGATAR motifs in GATA1-occupied sequences are highlighted. PBDE: peripheral blood-derived erythroblasts.

(d) G1E-ER-GATA1 cells were serum-starved, treated with or without bacterial sphingomyelinase (bSMase) for 30 min, and stimulated with SCF or Epo. SCF and Epo signaling were measured by Western blotting (Blots from 4 biologically independent samples were shown).

(e) Quantitation of blots in (d). p-AKT and p-STAT5 signals were normalized to their corresponding protein levels (n=4 biologically independent samples, mean ± SEM). p-values were calculated by two-tailed paired Student's t-test.

Source data are provided as a Source Data file.

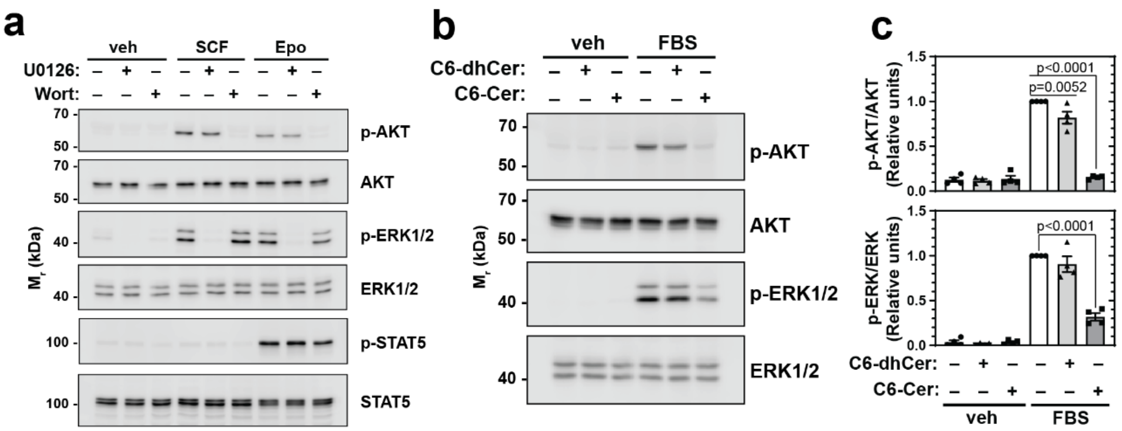

Supplementary Fig. 6. Ceramide homeostasis commissions AKT and ERK signaling post-receptor activation. Related to Fig. 6.

(a) G1E-ER-GATA1 cells were serum-starved, treated with U0126 or wortmannin for 1 h, and stimulated with SCF or Epo. SCF and Epo signaling were measured by Western blotting. Representative blots from 3 independent experiments were shown.

(b) G1E-ER-GATA1 cells were serum-starved, treated with C6-dhCer or C6-Cer for 30 min, and stimulated with FBS. AKT and ERK phosphorylation were measured by Western blotting.

(c) Quantitation of blots in (b). p-AKT and p-ERK signals were normalized to their corresponding protein levels (n=4 biologically independent samples, mean ± SEM). p-values were calculated using one-way ANOVA followed by Dunnett's multiple comparisons test.

Source data are provided as a Source Data file.

Supplementary Table 1. Primers for RT-qPCR

Primers	Species	Sequence (5'->3')
Eif3k mRNA	mouse	AGATGCTCGGAGACCTGACTG<br>CAATCTTCTCCACGATGTTCTTG
18S	mouse/<br>human	CGCCGCTAGAGGTGAAATTCT<br>CGAACCTCCGACTTTCGTTCT
Hbb-b1 mRNA	mouse	TTTAACGATGGCCTGAATCACTT<br>CAGCACAATCACGATCATATTGC
Hba-a1 mRNA	mouse	GTGGATCCCGTCAACTTCAAG<br>CAAGGTCACCAGCAGGCAGT
Slc4a1 mRNA	mouse	GGACAGATAGCATATAGAGACCTAACCA<br>CGTAGTCTGTGGCTGTTTGCTC
Alas2 mRNA	mouse	CCATCTTAAGGCAACCAAGGC<br>ACAGCATGAAAGGACAATGGC
Kit mRNA	mouse	AGCAATGGCCTCACGAGTTCTA<br>CCAGGAAAAGTTTGGCAGGAT
Epor mRNA	mouse	CTACAGCTTCTCATACCAGCTCGA<br>CTTAACCTAGTCTCCAGGCCCAT
Degs1 mRNA	mouse	TCTTGAAGGGACACGAAACC<br>GTACTCACTTGCGATCTTCCTC
Gata2 mRNA	mouse	GCAGAGAAGCAAGGCTCGC<br>CAGTTGACACACTCCCGGC
Cers2 mRNA	mouse	GCCACCCCATCCTCAATAAC<br>TCCTCATATCCTTTCTCCCCAG
Cers6 mRNA	mouse	CCATAGCCCTCAACATCCAAG<br>AATGCTCCGAACATCCCAG

Supplementary Table 2. Antibodies for Western blotting

ANTIBODY	SOURCE	IDENTIFIER
Phospho-Akt (S473) (D9E) XP® Rabbit mAb	Cell Signaling Technology	4060S; RRID:AB_2315049
Akt (pan) (C67E7) Rabbit mAb	Cell Signaling Technology	4691S; RRID:AB_915783
Phospho-p44/42 MAPK (Erk1/2) (Thr202/Tyr204) (D13.14.4E) XP® Rabbit mAb	Cell Signaling Technology	4370S; RRID:AB_2315112
p44/42 MAPK (Erk1/2) Rabbit Antibody	Cell Signaling Technology	9102S; RRID:AB_330744
Phospho-Stat5 (Tyr694) Rabbit Antibody	Cell Signaling Technology	9351S; RRID:AB_2315225
Stat5 (D2O6Y) Rabbit mAb	Cell Signaling Technology	94205S; RRID:AB_2737403
Phospho-Jak2 (Tyr1007/1008) Rabbit Antibody	Cell Signaling Technology	3771S; RRID:AB_330403
Jak2 (D2E12) XP® Rabbit mAb	Cell Signaling Technology	3230S; RRID:AB_2128522
Phospho-c-Kit (Tyr719) Rabbit Antibody	Cell Signaling Technology	3391S; RRID:AB_2131153
c-Kit (D13A2) XP® Rabbit mAb	Cell Signaling Technology	3074S; RRID:AB_1147633
β-Actin (8H10D10) Mouse mAb	Cell Signaling Technology	3700S; RRID:AB_2242334
Anti-MLD (DEGS1) Rabbit mAb [EPR9681]	Abcam	ab167169
GATA1 Rabbit Polyclonal Antibody	Bresnick lab	RRID:AB_2616055
GATA2 Rabbit Polyclonal Antibody	Bresnick lab	RRID:AB_2616054
Phospho-GATA1 (Ser310) Rabbit Polyclonal Antibody	Thermo Fisher Scientific	PA5-37582; RRID:AB_2554190
Anti-ORMDL3 Rabbit Polyclonal Antibody	MilliporeSigma	ABN417
I2PP2A (SET) Mouse mAb (F-9)	Santa Cruz Biotechnology	sc-133138; RRID:AB_2185628
Human Phospho-Erythropoietin R (Y426) Mouse mAb	R&D Systems	MAB6926; RRID:AB_10971652
Human EPOR Rabbit mAb	Wojchowski lab	N/A
Donkey anti-rabbit HRP-conjugated antibody	Jackson ImmunoResearch Laboratories	711-035-152 RRID:AB_10015282
Donkey anti-mouse HRP-conjugated antibody	Jackson ImmunoResearch Laboratories	715-035-150 RRID:AB_2340770

Supplementary Table 3. HPLC gradients for sphingolipid analysis

Gradient elution/time (min)	% MPB	% MPA
**(Dihydro)Ceramide/sphingoid bases**		
At injection	70	30
0 (after injection)-5	90	10
5-17	99	1
17-26	99	1
26-26.5	70	30
26.5-35	70	30

MPA: MS grade water containing 0.2% formic acid and 1 mM ammonium formate (pH 5.6)

MPB: MS grade methanol containing 0.2% formic acid and 1 mM ammonium formate (pH 5.6)

Supplementary Table 4. Conditions for mass spectrometry.

**Equipment**	Thermo Scientific Quantiva triple quadrupole mass spectrometer (Thermo Fisher Scientific, Waltham, MA, USA)
**Mode**	Electrospray Ion Source operating in positive ion Multiple Reaction Monitoring (MRM)
**Vaporizer Temperature**	400°C
**Capillary Temperature**	350°C
**Spray Voltage**	3500V
**Sheath**	60
**Ion sweep**	0
**Auxillary gases**	15
