# Supplementary material for: A transcriptional network governing ceramide homeostasis establishes a cytokine-dependent developmental process
Source: Nat Commun. 2023 Nov 9;14:7262. doi: 10.1038/s41467-023-42978-w (PMC10636182; doi:10.1038/s41467-023-42978-w)
Supplement: Supplementary file 3 — Reporting Summary [file 41467_2023_42978_MOESM3_ESM.pdf]

nature portfolio

Corresponding author(s): Emery H. Bresnick

Last updated by author(s): Oct 10, 2023

Reporting Summary

Nature Portfolio wishes to improve the reproducibility of the work that we publish. This form provides structure for consistency and transparency in reporting. For further information on Nature Portfolio policies, see our Editorial Policies and the Editorial Policy Checklist.

Statistics

For all statistical analyses, confirm that the following items are present in the figure legend, table legend, main text, or Methods section.

n/a	Confirmed	
☐	☒	The exact sample size (*n*) for each experimental group/condition, given as a discrete number and unit of measurement
☐	☒	A statement on whether measurements were taken from distinct samples or whether the same sample was measured repeatedly
☐	☒	The statistical test(s) used AND whether they are one- or two-sided<br>*Only common tests should be described solely by name; describe more complex techniques in the Methods section.*
☐	☒	A description of all covariates tested
☐	☒	A description of any assumptions or corrections, such as tests of normality and adjustment for multiple comparisons
☐	☒	A full description of the statistical parameters including central tendency (e.g. means) or other basic estimates (e.g. regression coefficient) AND variation (e.g. standard deviation) or associated estimates of uncertainty (e.g. confidence intervals)
☐	☒	For null hypothesis testing, the test statistic (e.g. *F*, *t*, *r*) with confidence intervals, effect sizes, degrees of freedom and *P* value noted<br>*Give P values as exact values whenever suitable.*
☒	☐	For Bayesian analysis, information on the choice of priors and Markov chain Monte Carlo settings
☒	☐	For hierarchical and complex designs, identification of the appropriate level for tests and full reporting of outcomes
☒	☐	Estimates of effect sizes (e.g. Cohen's *d*, Pearson's *r*), indicating how they were calculated

Our web collection on statistics for biologists contains articles on many of the points above.

Software and code

Policy information about availability of computer code

Data collection	ImageStudio v5.2, QuantStudio v1.6.1,
Data analysis	GraphPad Prism 7, ImageStudio Lite v5.2, QuantStudio v1.6.1, FlowJo v10.1, LipiDex, Compound Discoverer 2.0.

For manuscripts utilizing custom algorithms or software that are central to the research but not yet described in published literature, software must be made available to editors and reviewers. We strongly encourage code deposition in a community repository (e.g. GitHub). See the Nature Portfolio guidelines for submitting code & software for further information.

Data

Policy information about availability of data

All manuscripts must include a data availability statement. This statement should provide the following information, where applicable:

- Accession codes, unique identifiers, or web links for publicly available datasets
- A description of any restrictions on data availability
- For clinical datasets or third party data, please ensure that the statement adheres to our policy

The discovery lipidomics data generated in this study have been deposited to MassIVE database under accession code MSV000092894 (https://massive.ucsd.edu/ProteoSAFe/dataset.jsp?task=e77cdb5b6322473ba9b27b0df992c75d). All other data generated in this study are provided in the paper or Supplementary Information/Source Data file. Source data are provided with this paper.

Research involving human participants, their data, or biological material

Policy information about studies with human participants or human data. See also policy information about sex, gender (identity/presentation), and sexual orientation and race, ethnicity and racism.

Reporting on sex and gender	Deidentified human cells were used. Information on sex and gender is not available.
Reporting on race, ethnicity, or other socially relevant groupings	Deidentified human cells were used. Information on race, ethnicity, or other socially relevant groupings is not available.
Population characteristics	Deidentified human cells were used. Information on population characteristics is not available.
Recruitment	Deidentified human cells were used. Information on recruitment is not available.
Ethics oversight	Deidentified human cells that are exempt from human subject regulations were used. The cells were handled according to approved biosafety protocol B00000056-AM008 at University of Wisconsin-Madison.

Note that full information on the approval of the study protocol must also be provided in the manuscript.

Field-specific reporting

Please select the one below that is the best fit for your research. If you are not sure, read the appropriate sections before making your selection.

[x] Life sciences [ ] Behavioural & social sciences [ ] Ecological, evolutionary & environmental sciences

For a reference copy of the document with all sections, see nature.com/documents/nr-reporting-summary-flat.pdf

Life sciences study design

All studies must disclose on these points even when the disclosure is negative.

Sample size	No sample size calculation was performed. Sample sizes were chosen based on experience and standard protocol. The sample sizes were sufficient as they give reliable and reproducible signals in a given experiment.
Data exclusions	No data were excluded from the analyses.
Replication	All experiments were replicated or performed independently at least 3 times. All attempts at replication were successful.
Randomization	Randomization was not relevant to our study since all experiments were based on established cell lines and primary cells.
Blinding	Investigators were blinded to group allocation for colony forming unit assays and cell viability assays. For other assays, blinding was not possible because the samples need to be loaded in a specific order.

Reporting for specific materials, systems and methods

We require information from authors about some types of materials, experimental systems and methods used in many studies. Here, indicate whether each material, system or method listed is relevant to your study. If you are not sure if a list item applies to your research, read the appropriate section before selecting a response.

Materials & experimental systems

n/a	Involved in the study	
[ ]	[x]	Antibodies
[ ]	[x]	Eukaryotic cell lines
[x]	[ ]	Palaeontology and archaeology
[ ]	[x]	Animals and other organisms
[x]	[ ]	Clinical data
[x]	[ ]	Dual use research of concern
[x]	[ ]	Plants

Methods

n/a	Involved in the study	
[x]	[ ]	ChIP-seq
[ ]	[x]	Flow cytometry
[x]	[ ]	MRI-based neuroimaging

Antibodies

Antibodies used	Antibodies used are listed in Supplementary Table 2.
Validation	Antibodies against p-AKT, AKT, p-ERK1/2, ERK1/2, p-STAT5, STAT5, p-KIT, KIT and beta-actin from Cell Signaling Technologies were validated for Western blotting in human and mouse according to statements on the manufacturer's website. Antibody against DEGS1

from Abcam was validated for Western blotting in human and mouse according to manufacturer's statement and data provided in the manuscript (Fig. 1e). Antibodies against GATA1 and GATA2 from Bresnick lab were validated for Western blotting in mouse in previous publication (Im et al., PNAS, 2005). Antibody against p-GATA1 from ThermoFisher Scientific was validated for Western blotting in human and mouse according to manufacturer's statement. Antibody against ORMDL3 from MilliporeSigma was validated for Western blotting in human and mouse according to manufacturer's statement and data provided in the manuscript (Fig. 5n). Antibody against SET from Santa Cruz was validated for Western blotting in human and mouse according to manufacturer's statement and data provided in the manuscript (Fig. 5d). Antibody against p-EPOR from R&D Systems was validated for Western blotting in human and mouse according to manufacturer's statement and data provided in the manuscript (Fig. 4k,m and Fig. 6m). Antibody against EPOR from Wojchowski lab (University of New Hampshire) was validated for Western blotting in human and mouse according to previous publication (Singh et al, PLoS One, 2012) and data provided in the manuscript (Fig. 4k,m and Fig. 6m).

Eukaryotic cell lines

Policy information about cell lines and Sex and Gender in Research

Cell line source(s)	G1E-ER-GATA1 cells derived from GATA1-mutant male murine ES cells were obtained from Mitchell J. Weiss lab (St. Jude). hi-WT cells were derived from mouse fetal liver hematopoietic progenitor cells by Emery Bresnick lab. Primary human mononuclear cells were isolated from G-CSF-mobilized peripheral blood from donors at University of Wisconsin-Madison. The sex of the primary cells used for experiments is not available.
Authentication	None of the cell lines used were authenticated.
Mycoplasma contamination	The cell lines were not tested for mycoplasma contamination.
Commonly misidentified lines (See ICLAC register)	No commonly misidentified lines were used in this study.

Animals and other research organisms

Policy information about studies involving animals; ARRIVE guidelines recommended for reporting animal research, and Sex and Gender in Research

Laboratory animals	Mus musculus C57BL/6J. Fetal livers from E14.5 mouse embryos were used in this study.
Wild animals	No wild animals were used in this study.
Reporting on sex	Sex was not considered in study design.
Field-collected samples	No field-collected samples were used in this study.
Ethics oversight	All animals were handled according to approved institutional animal care and use committee (IACUC) protocols (#M02230) of the University of Wisconsin-Madison. All animal experiments were performed with the ethical approval of the AAALAC International (Association for the Assessment and Accreditation of Laboratory Animal Care) at the University of Wisconsin-Madison.

Note that full information on the approval of the study protocol must also be provided in the manuscript.

Flow Cytometry

Plots

Confirm that:

- [x] The axis labels state the marker and fluorochrome used (e.g. CD4-FITC).
- [x] The axis scales are clearly visible. Include numbers along axes only for bottom left plot of group (a 'group' is an analysis of identical markers).
- [x] All plots are contour plots with outliers or pseudocolor plots.
- [x] A numerical value for number of cells or percentage (with statistics) is provided.

Methodology

Sample preparation	For apoptosis analysis, G1E-ER-GATA1 cells or primary human erythroblasts were washed with PBS and then washed with Annexin V binding buffer (10 mM HEPES, 140 mM NaCl, 2.5 mM CaCl2, pH 7.4). Cells were stained with Alexa Fluor® 647-Annexin V (BioLegend, 640912, 1:40) in 100 µl Annexin V binding buffer and incubated at RT for 15 min. Cells were then washed with 1 ml Annexin V binding buffer and resuspended in 500 µl Annexin V binding buffer + 1 µg/ml DAPI before analysis.<br>For erythroid differentiation assay, G1E-ER-GATA1 or primary mouse fetal liver cells were washed with PBS + 2% FBS and stained with PE-CD71 (BioLegend, 113808, 1:100) and APC-Ter119 (BioLegend, 116212, 1:100) for 30 min on ice. Cells were then washed with 2% FBS, 10 mM glucose and 2.5 mM EDTA in PBS.
Instrument	Attune™ NxT Flow Cytometer (Thermo Fisher Scientific)

Software	FlowJo v10.1
Cell population abundance	N/A
Gating strategy	Single cells were gated using FSC-A, FSA-H, SSC-A, SSC-H. Live cells were gated by DAPI-negative staining. Single fluorophore-stained samples were used to define positive and negative staining populations.

☒ Tick this box to confirm that a figure exemplifying the gating strategy is provided in the Supplementary Information.
