# Supplementary material for: A transcriptional network governing ceramide homeostasis establishes a cytokine-dependent developmental process
Source: Nat Commun. 2023 Nov 9;14:7262. doi: 10.1038/s41467-023-42978-w (PMC10636182; doi:10.1038/s41467-023-42978-w)
Supplement: Supplementary file 4 — Description of Additional Supplementary Files [file 41467_2023_42978_MOESM4_ESM.pdf]

## Description of Additional Supplementary files

File name: Supplementary Data 1.
Description: Quantitative lipidomics for sphingolipds
Undifferentiated (-E2) or differentiated (+E2) G1E-ER-GATA1 cells were treated with or without 4HPR for 24 or 48 h and the levels of sphingolipids were quantified by lipidomics using internal standards and normalized to inorganic phosphate (Pi).

File name: Supplementary Data 2.
Description: Discovery lipidomics WT or mutant (Alas2-enhancer deleted) G1EER-GATA1 cells were undifferentiated (-E2) or differentiated (+E2) for 48 h and the levels of lipids were quantified by relative quantitation lipidomics.

File name: Supplementary Data 3.
Description: Transitions and retention time for quantitative lipidomics
The mass transitions and retention time for each sphingolipid species detected and internal standard used are listed.
