# Supplementary material for: A transcriptional network governing ceramide homeostasis establishes a cytokine-dependent developmental process
Source: Nat Commun. 2023 Nov 9;14:7262. doi: 10.1038/s41467-023-42978-w (PMC10636182; doi:10.1038/s41467-023-42978-w)

Fig. 1e

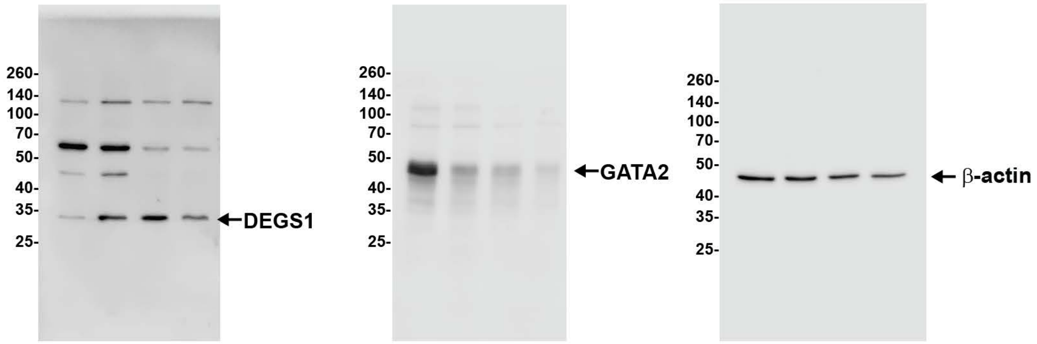

Fig. 4b

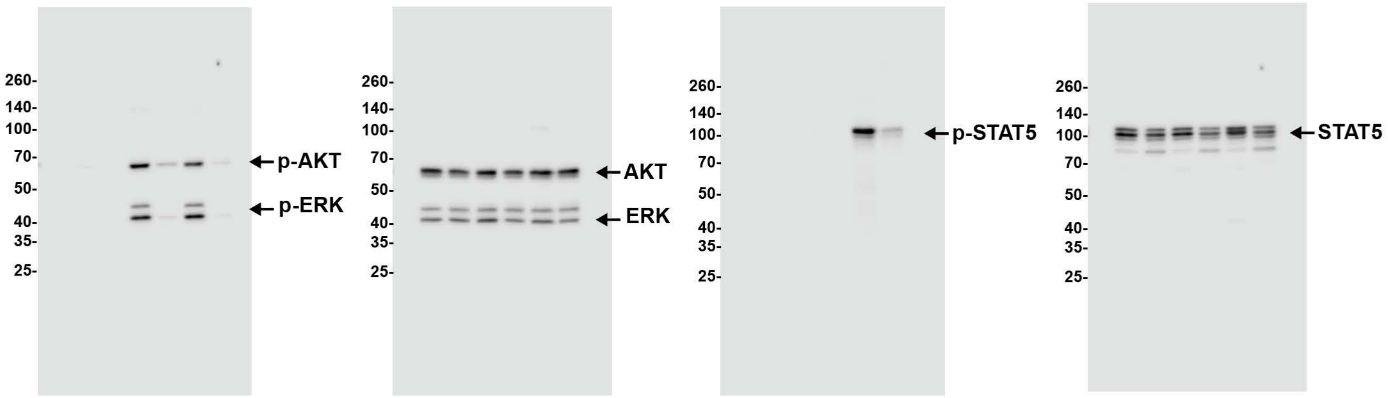

Fig. 4e

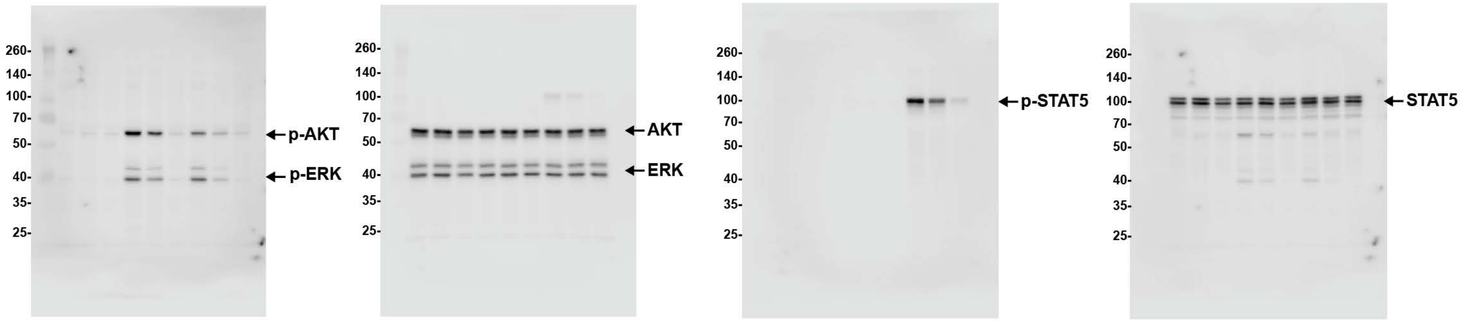

Fig. 4h

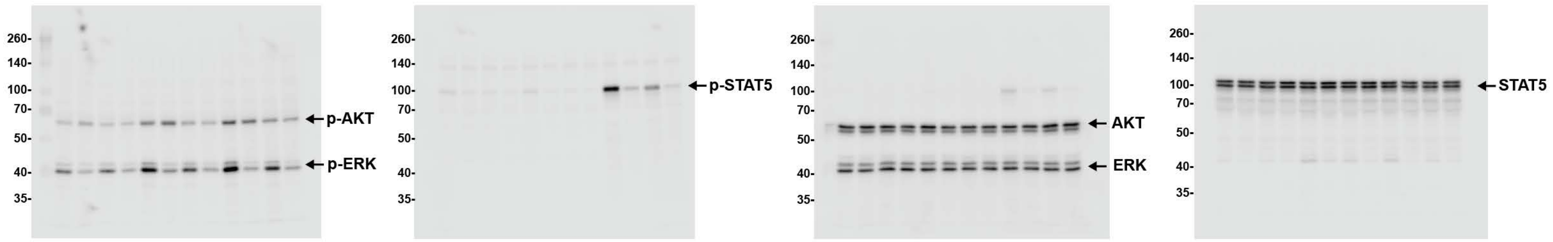

Fig. 4j

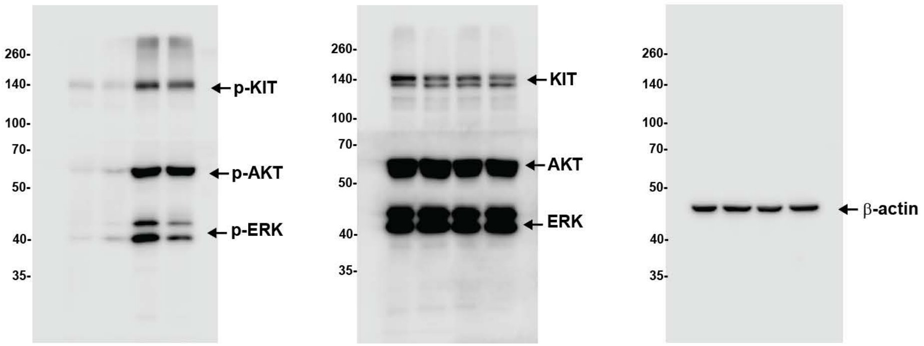

Fig. 4k

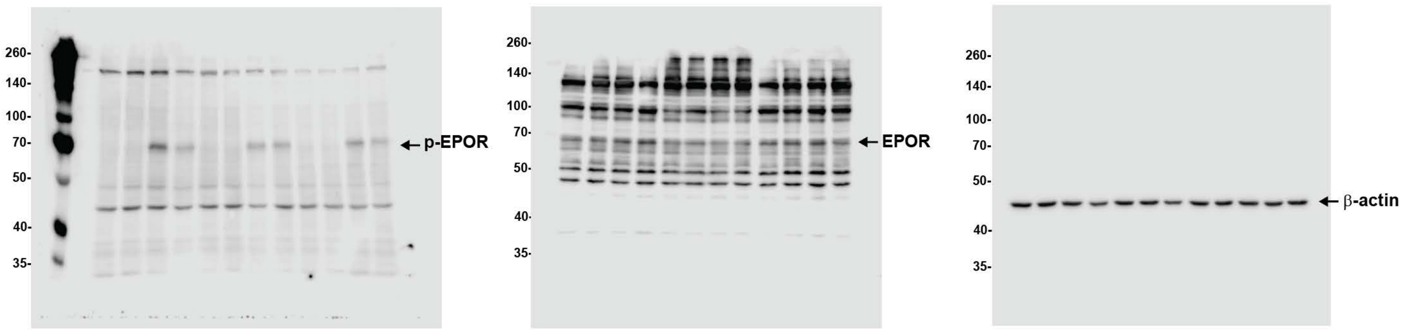

Fig. 4l

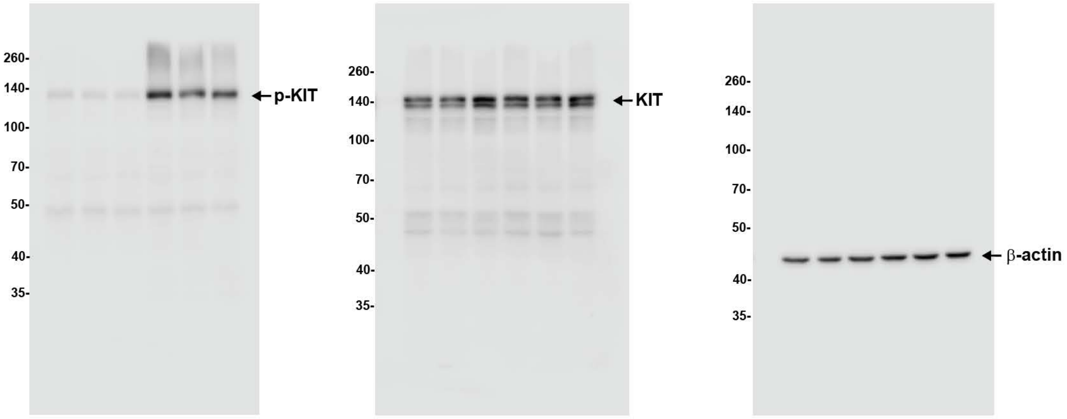

Fig. 4m

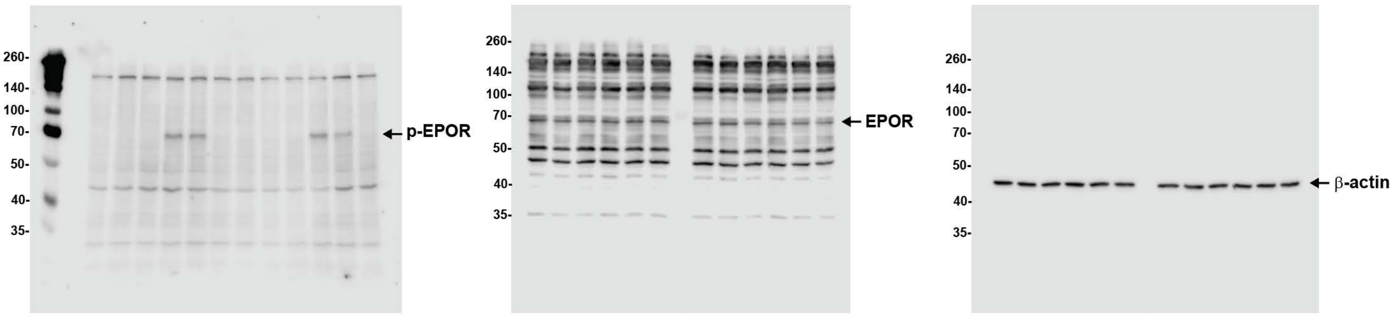

Fig. 5a

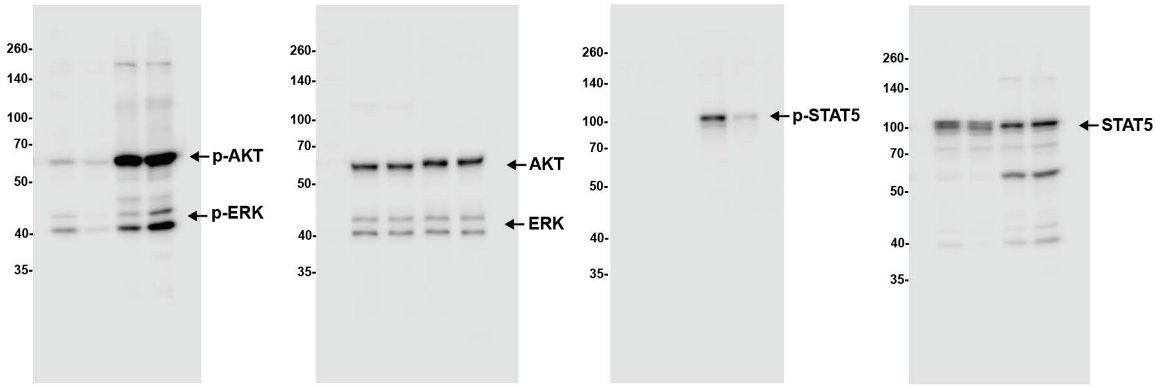

Fig. 5b

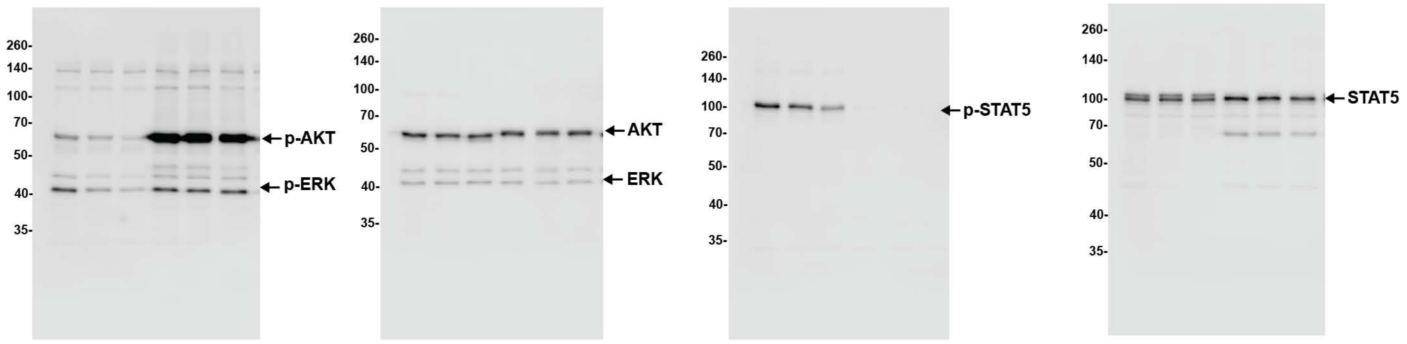

Fig. 5d

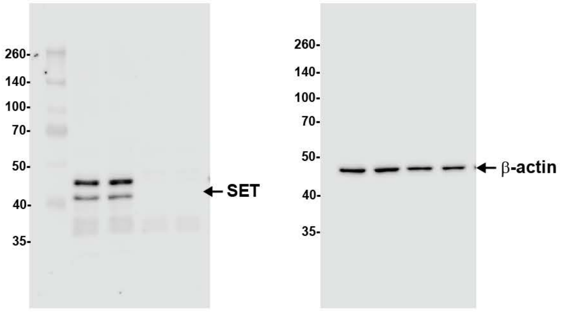

Fig. 5e

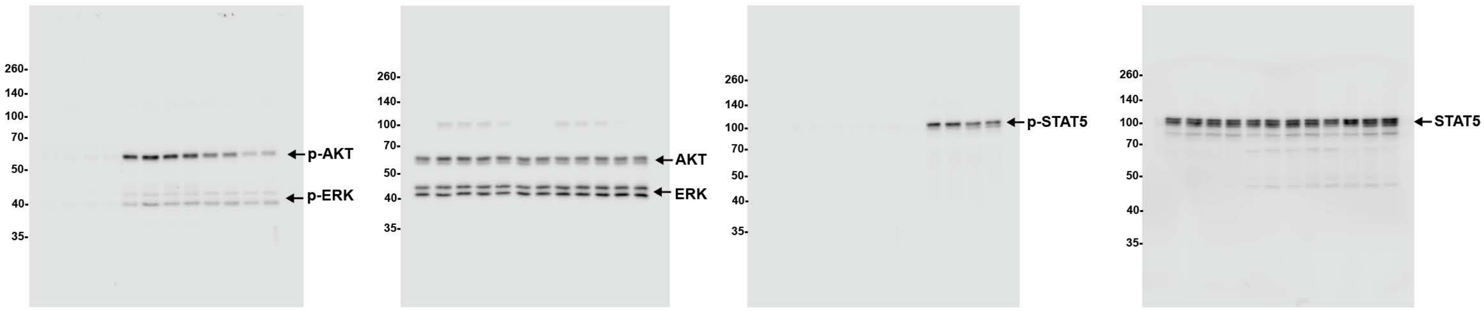

Fig. 5g

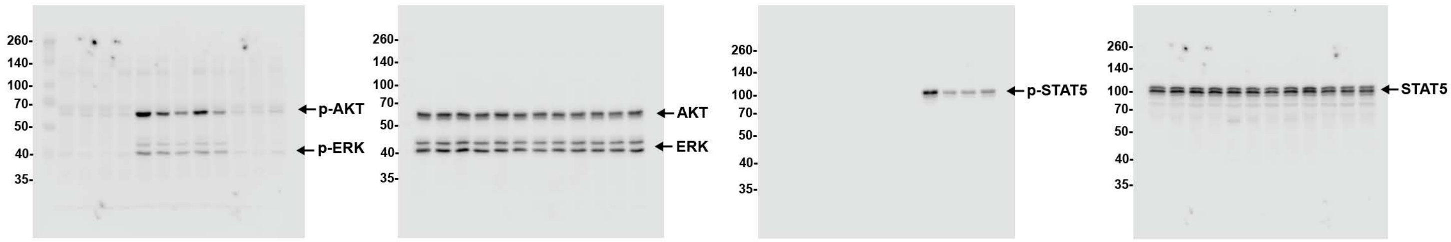

Fig. 5i

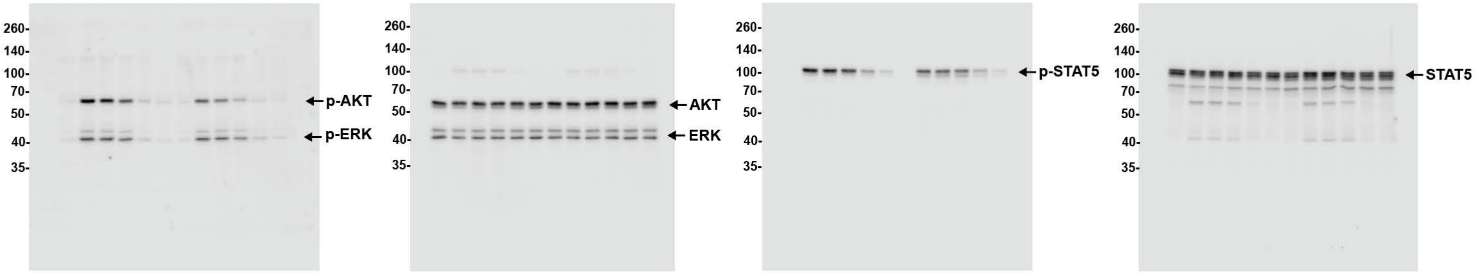

Fig. 5n

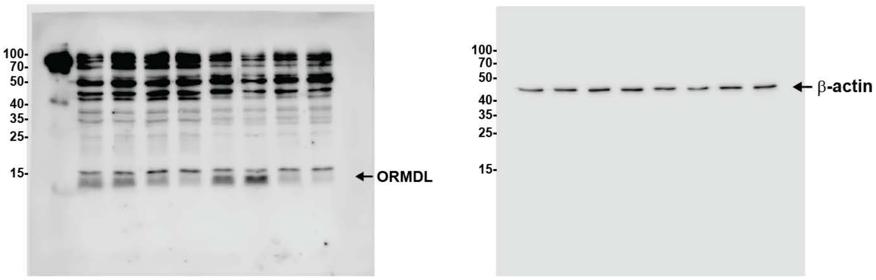

Fig. 5o

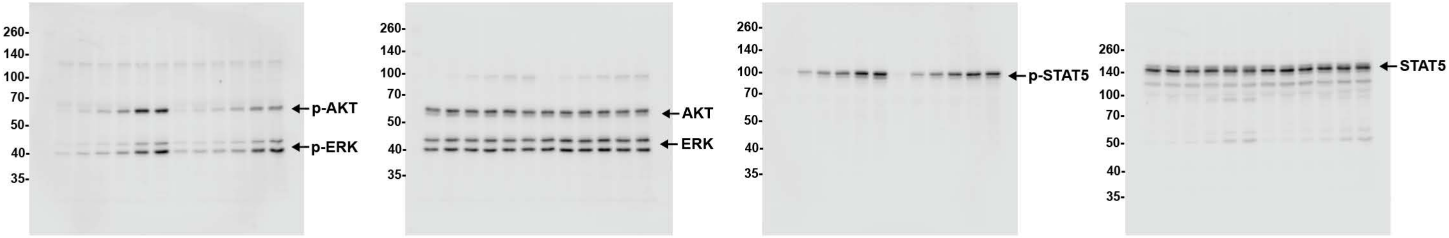

Fig. 6a

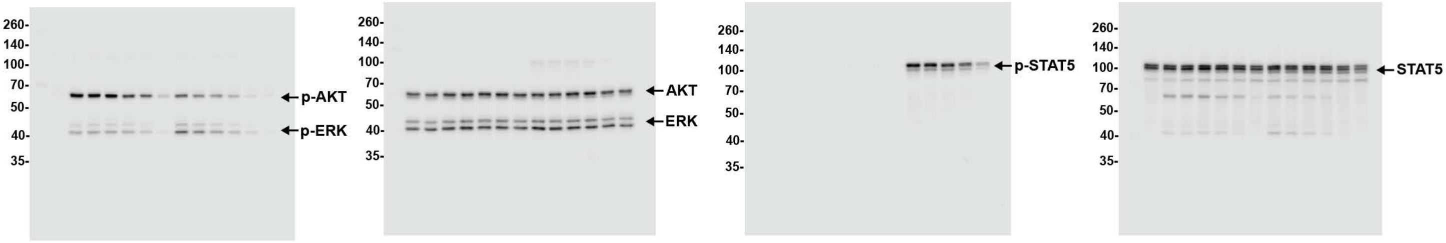

Fig. 6c

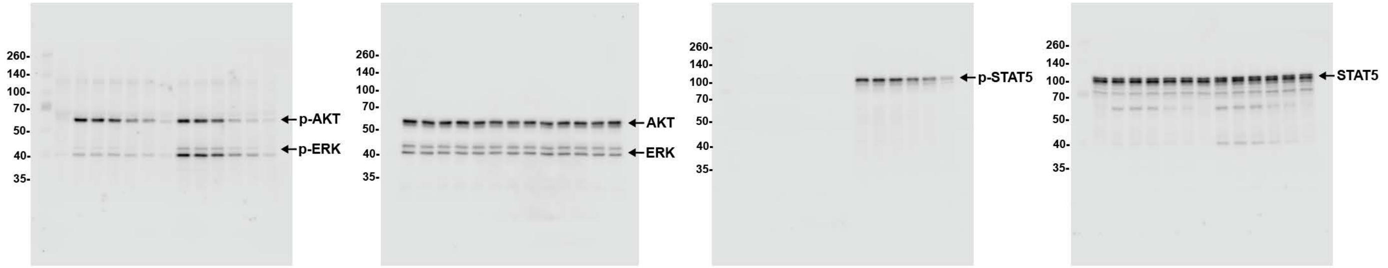

Fig. 6e

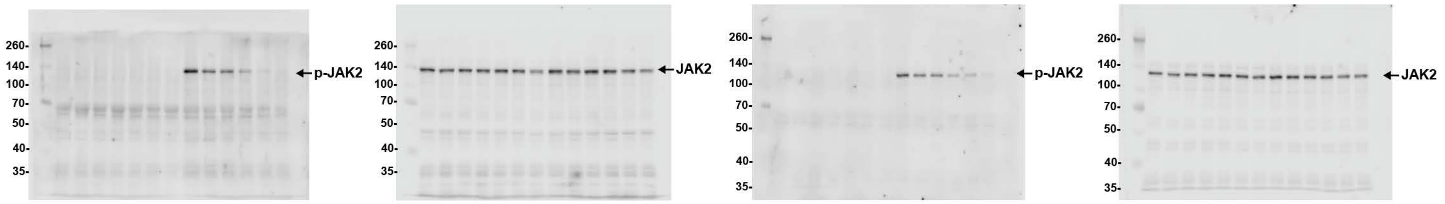

Fig. 6f

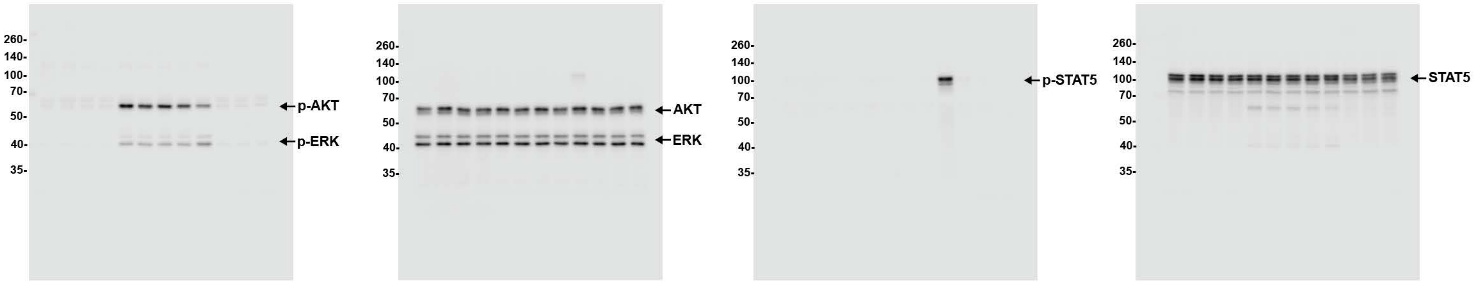

Fig. 6g

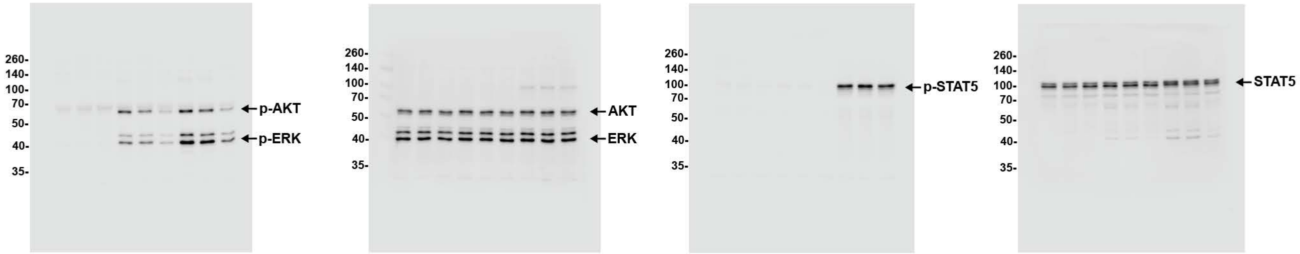

Fig. 6i

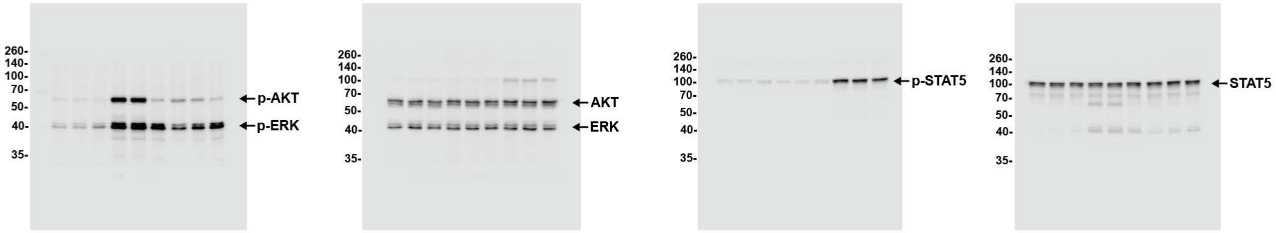

Fig. 6k

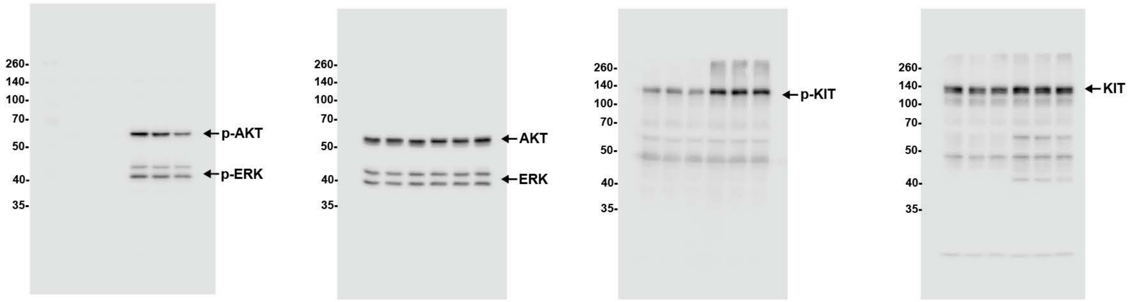

Fig. 6m

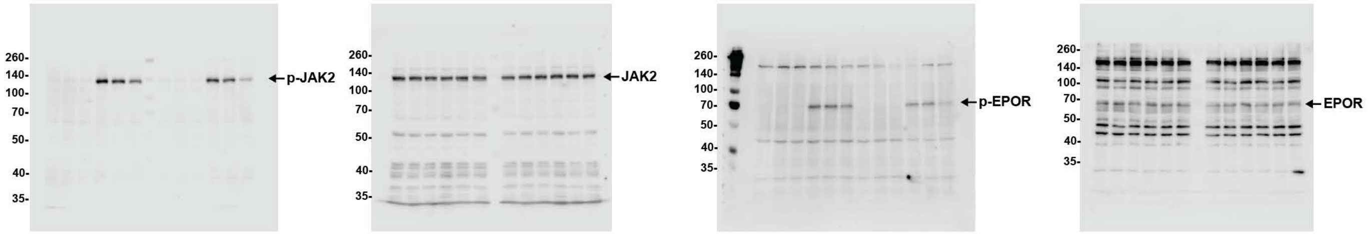

Supplementary Fig. 4a

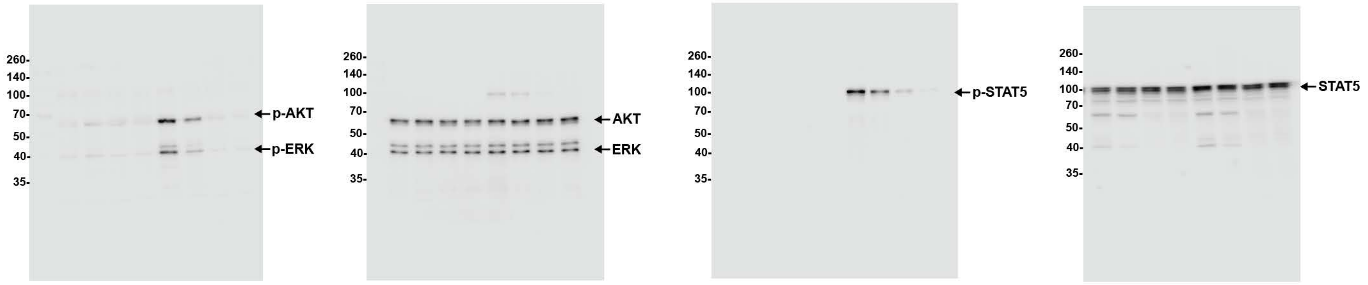

Supplementary Fig. 4d

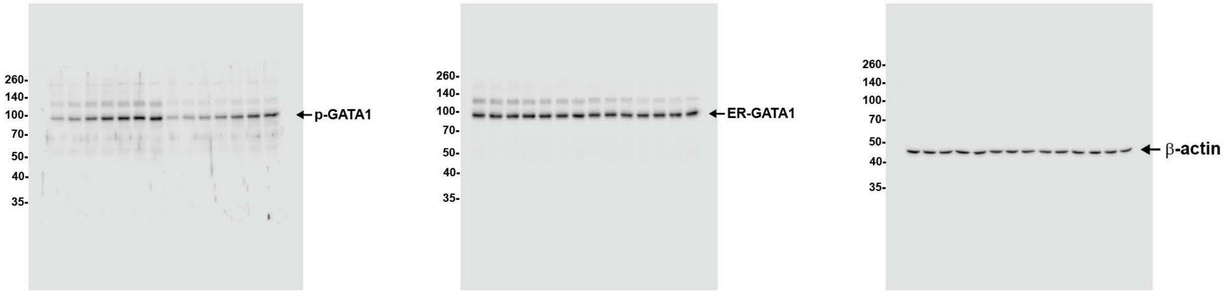

Supplementary Fig. 5d

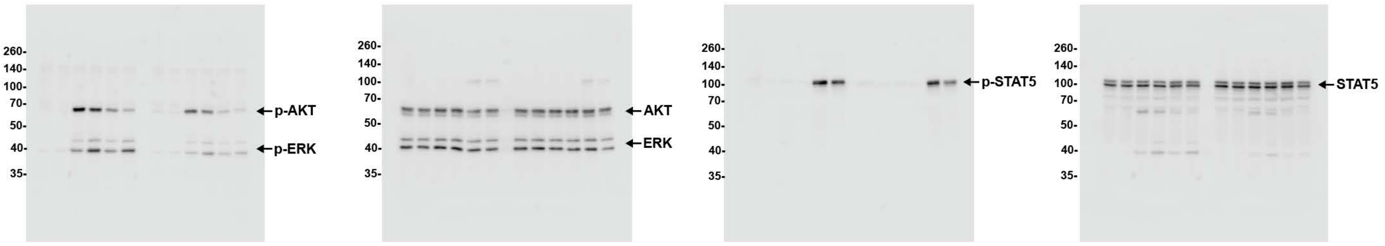

Supplementary Fig. 6a

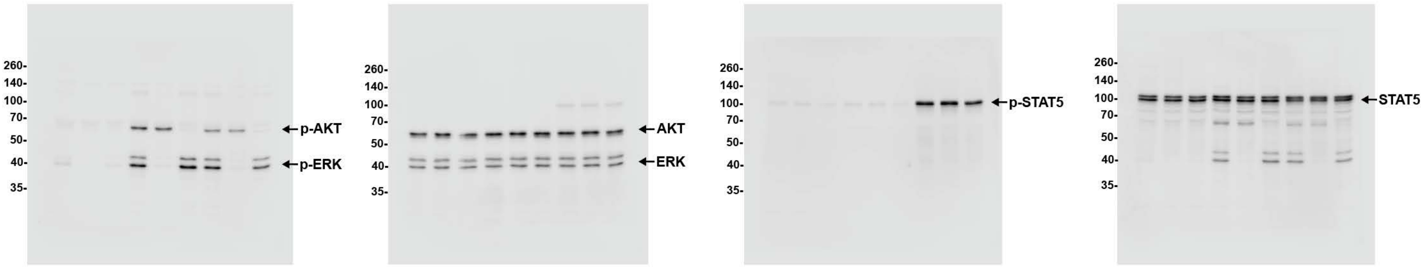

Supplementary Fig. 6b

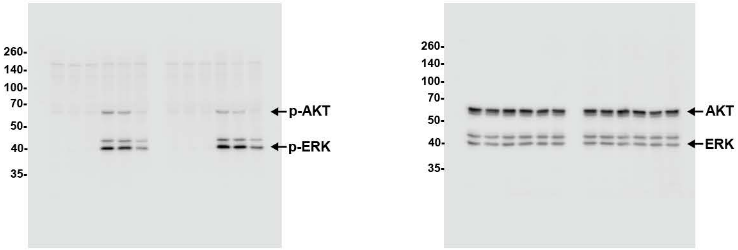

Supplement: Supplementary file 8 — Source Data [file 41467_2023_42978_MOESM8_ESM.zip › Source Data/Source Data File_Uncropped Images.pdf]
